# Social capital and transaction costs in millet markets

**DOI:** 10.1016/j.heliyon.2018.e00505

**Published:** 2018-01-12

**Authors:** Damien Christophe Jacques, Eduardo Marinho, Raphaël d'Andrimont, François Waldner, Julien Radoux, Frédéric Gaspart, Pierre Defourny

**Affiliations:** a Earth and Life Institute, Université Catholique de Louvain, 1348, Louvain-la-Neuve, Belgium; b Rio de Janeiro, Brazil; c CSIRO Agriculture & Food, Australia

**Keywords:** Economics

## Abstract

In sub-Saharan Africa, transaction costs are believed to be the most significant barrier that prevents smallholders and farmers from gaining access to markets and productive assets. In this study, we explore the impact of social capital on millet prices for three contrasted years in Senegal. Social capital is approximated using a unique data set on mobile phone communications between 9 million people allowing to simulate the business network between economic agents. Our approach is a spatial equilibrium model that integrates a diversified set of data. Local supply and demand were respectively derived from remotely sensed imagery and population density maps. The road network was used to establish market catchment areas, and transportation costs were derived from distances between markets. Results demonstrate that accounting for the social capital in the transaction costs explained 1–9% of the price variance depending on the year. The year-specific effect remains challenging to assess but could be related to a strengthening of risk aversion following a poor harvest.

## 1 Introduction

In sub-Saharan Africa, the functioning of food markets is jeopardized by several barriers that prevent smallholders and farmers from gaining access to markets and productive assets. The most significant of these barriers is believed to be the transaction costs, the observable and hidden costs associated with arranging and carrying out a transaction. The role that social capital might play in shaping these costs is a research question that has captured the attention of many during the last two decades (Durlauf and Fafchamps, 2005). In this paper, we refer to the concept of social capital as introduced by Putnam et al. (1994), that is, the “features of social organization, such as trust, norms, and networks that can improve the efficiency of society” (p. 167).

Social capital can lower transactions costs by, *e.g.*, reducing the information and search costs, increasing trust or cutting down the administrative burden (Fafchamps, 2006; Fafchamps and Minten, 2001; Fukuyama, 1995; Knack and Keefer, 1997; Woolcock and Narayan, 2000). If agents are not well informed about price differences across markets, time periods or buyers and sellers of different types, or if such information is asymmetric, they cannot engage in optimal arbitrage (Tollens, 2006). On the other hand, trust helps to mitigate the abuse that can occur during the purchase and sale of commodities (non-delivery, late payment, deficient quality, incorrect quantity...) (Bigsten et al., 2000). As they can more easily find and screen each other, well-connected agents will also be more likely to trade together (Barr, 2000). However, the effect is not necessarily positive as overreliance in the activities and decisions of relatives can lead to overpricing due to traders' errors (Levine et al., 2014; Portes, 2014). Nevertheless, limited information and mistrust generally results in inefficient transmission of prices due to local surpluses or scarcities, which ultimately affects both producers and consumers.

According to Durlauf and Fafchamps (2005), the literature on the effects of social capital can be divided into individual and aggregate studies. On the one hand, individual studies explore the effect of social capital on some individual outcomes. For instance, Fafchamps and Minten (2002) found a significant effect of social capital on total sales of food traders in Madagascar, Mawejje and Terje Holden (2014) highlighted that social capital can help Ugandan household to receive higher prices for coffee and Grootaert (1999) demonstrated the effect of social capital household expenditure in Indonesia. On the other hand, aggregate studies mainly focused on the relationship between social capital and per capita output growth at a high level of aggregation, *e.g.*, a country or a region (Beugelsdijk and Schaik, 2003; Guiso et al., 2004). The standard approach of all these studies is generally to run linear regressions on cross-sectional data with some outcome of interest against empirical proxies for social capital and a set of controls. The significance of the coefficients of the social capital variables allows to conclude on their effect on the outcome. One challenge of empirical work on social capital is therefore to identify observable variables that can be used as proxies for social capital (Portes, 2000). An array of variables have been proposed in empirical papers and include the number of known traders, the number of relatives involved in agricultural trade, the number of languages the trader speaks, or some measures of ethnic homogeneity for organizations formed by households (Fafchamps and Minten, 2001, Fafchamps and Minten, 2002; Gabre-Madhin, 2001; Grootaert, 1999; Isham, 2002).

In this study, we explored the effect of transaction costs generated by social capital on millet retail prices in Senegalese food markets for three contrasted years. Millet serves as the main local subsistence food crops in many Sahelian countries, including Senegal. Millet prices are therefore an important indicator of food security as they directly impact farmers' income and their ability to access staple foods (Pokhriyal and Jacques, 2017). Furthermore, millet is an interesting case study as most trade is local (little cross-border trade). We modeled social capital using a unique data set of mobile phone communications between 9 million people. Our assumption is that the intermarket calls reflects the business network of economic agents from different markets. Other things being equal, well-connected agents are more likely to trade with one another because the transaction costs are reduced between them. To evaluate the effect of social capital on millet prices and market functioning, we focused on intermarket trades as traders are the economic agents most exposed to the effect of transaction costs (Fafchamps and Minten, 2001). To that end, we adopted an original approach in the form of a spatial equilibrium model which enabled us to compare different market functioning scenarios, *i.e.*, with and without transaction costs accounting for social capital. Local supply and demand were respectively derived from remotely sensed imagery and population density maps. The road network was used to establish market catchment areas, and transportation costs were derived from distances between markets. The emphasis was on the parsimony of the model by making use of available data sets without seeking to determine the eventual causal links of the mechanisms involved.

We showed that taking into account the impact of social capital on transaction costs explained between 1 and 9 percent of the price variance depending on the year. The year-specific effect remains challenging to assess but could be related to a strengthening of risk aversion following a poor harvest. In any case, the high difference between the years suggests that the effect of social capital in agricultural markets is very dynamic and context specific.

The remainder of the paper is organized as follows: Section 2 describes the empirical framework. Section 3 presents the data, Section 4 provides the findings and discussions as well as the limitations of the approach and some policy implications. Lastly, Section 5 provides the main conclusions of the analysis.

## 2 Model

To assess the effect of social capital on transaction costs, we used a simple spatial equilibrium model. Specifically, a point-location model consisting of a network with markets located at network nodes, and network links that serve only for commodity transportation flows (Enke, 1951; McNew and Fackler, 1997; Takayama and Judge, 1971), which differs from the agents-on-links models (Hotelling, 1990). In a given market, *i*, the price is a function of local supply, *S*, demand, *D* and time, *t* (Eq. (1)):

(1) $$p_{i}=f(S_{i},D_{i},t)$$

Each pair of market nodes is linked by trade. Without transaction costs, price differences between markets are only depending on the transportation cost, *r* (assumed to be identical throughout the country), and the distance between market *i* and *j*, $d_{ij}$ (Eq. (2)):

(2) $$p_{i}-p_{j}\leq d_{ij}r$$

with equality if trading actually occurs.

We introduced a transaction cost, associated with social capital, as the multiplicative parameter $\frac{1}{s}$. For each pair of markets *ij* and depending on the level of social capital, the transaction cost was either null ($s_{ij}=1$) or infinite ($s_{ij}=0$). It followed that the arbitrage condition became Eq. (3):

(3) $$p_{i}-p_{j}\leq \frac{d_{ij}r}{s_{ij}}\;\text{with}\;s_{ij}\in \{0,1\}$$

Through an optimization procedure, the two unknown parameters, *r* and *s*, were estimated. The objective was to assess if Eq. (3) allowed to explain more of the price variance than Eq. (2), *i.e.*, if taking into account the impact of social capital on transaction costs allowed to better explain price variance.

In a nutshell, three scenarios were compared: (i) r=∞ (Scenario I – segregated markets), (ii) 0<r<∞ and $\overset{¯}{s}=1$ (Scenario II – trade without transaction costs) and (iii) 0<r<∞ and $0<\overset{¯}{s}\leq 1$ (Scenario III – trade with transaction costs) with $\overset{¯}{s}$, the mean of all $s_{ij}$.

**Figure 1:**
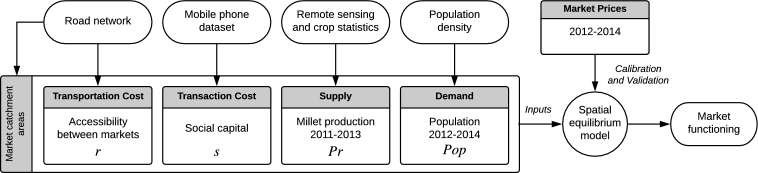
Overview of the data inputs and their associated variables.

**Figure 2:**
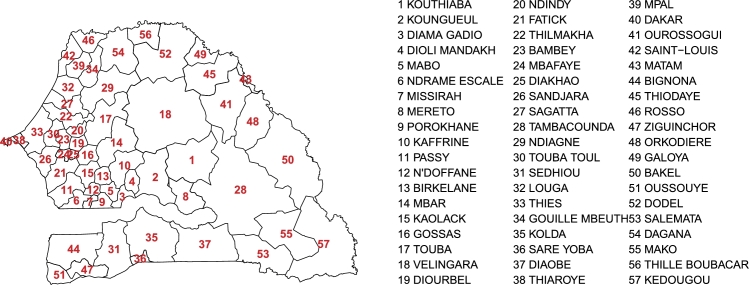
Catchment areas of each market.

Our approach (Figure 1) involved estimating the retail price for millet in each market from estimation of local demand and supply (Eq. (1)). Population was used as a proxy of demand. Local food production was approximated by a vegetation index derived from satellite image time series combined with national statistics and used as the supply input. Only local production was considered as millet is little affected by international trade. Both types of data were spatially aggregated in the catchment area of each market, which equates to the area that minimizes road distance between each market (Figure 2).

### 2.1 Scenario I (segregated markets)

For segregated markets, the price was estimated using the following multiple regression model (ordinary least squares) fitted separately for each year (Eq. (4)):

(4) $$p_{it}=\alpha_{0}+\alpha_{1}log\left(\frac{Pop_{i}}{Pr_{i}^{init}}\right)+\alpha_{2}M_{t}+\epsilon_{it}$$

where $Pop_{i}$ and $Pr_{i}^{init}$ are the population and the millet production (in tons) of the catchment area of the market *i*, *M*, the month and $\epsilon_{it}$, the regression residual. We restricted the analysis from January to August. This corresponds to the period following harvest up to the lean season, and is regarded as the most critical period affecting price evolution.

### 2.2 Scenario II and Scenario III (spatial equilibrium model)

One could use Eq. (4) to estimate the market prices within the spatial equilibrium model. However, there is no reason to believe that a model fitted on unrealistic distribution of production ($Pr^{init}$) would be appropriate to simulate trading. To overcome this limitation, we introduced pseudo prices, proportional to actual prices, defined as Eq. (5):

(5) $$p_{it_{0}}\propto {\overset{˜}{p}}_{it_{0}}=log\left(\frac{Pop_{i}}{Pr_{i}}\right)$$

The initial conditions of the spatial equilibrium model were the market prices estimated by Eq. (5) with $Pr_{i}=Pr_{i}^{init}$. All production transfers were assumed to occur in $t_{0}$ so that the month variable *M* was not used in the spatial equilibrium model. The arbitrage condition defined in Eq. (3) was then applied to each pair of markets with the transportation cost *r* set as the transportation pseudo cost $\overset{˜}{r}\propto r$. If an opportunity of arbitrage was possible between market *i* and *j*, *i.e.*, if the condition defined in Eq. (3) was not satisfied, a ton of millet was transferred between the two markets. The new level of production in each market then allowed estimation of new prices (using Eq. (5)) on which the condition of transfer was again applied. Through this approach, the model iterates until an equilibrium was reached, *i.e.*, when all profitable transfers of production between markets had occurred and Eq. (3) was satisfied for all pair of markets. The only effect of the model was the reallocation of production between the markets. The total production remained unchanged.

Actual market prices were then estimated by fitting the following model at the equilibrium (Eq. (6)):

(6) $$p_{it\overset{˜}{r}\overset{¯}{s}}=\gamma_{0,\overset{˜}{r}\overset{¯}{s}}+\gamma_{1,\overset{˜}{r}\overset{¯}{s}}log\left(\frac{Pop_{i}}{Pr_{i\overset{˜}{r}\overset{¯}{s}}^{eq}}\right)+\gamma_{2,\overset{˜}{r}\overset{¯}{s}}M_{t}+\mu_{it\overset{˜}{r}\overset{¯}{s}}$$

where $Pr_{i\overset{˜}{r}\overset{¯}{s}}^{eq}$ is the production at the equilibrium of market *i* obtained for a specific pair of parameters $\overset{˜}{r}$ and $\overset{¯}{s}$ (mean of all $s_{ij}$), and this was the only variable that changes compared to Eq. (4). This approach was repeated with several values of $\overset{˜}{r}\in \left[min\left(\frac{\Delta {\overset{˜}{p}}_{ij}^{init}}{d_{ij}}\right),max\left(\frac{\Delta {\overset{˜}{p}}_{ij}^{init}}{d_{ij}}\right)\right]$ and $\overset{¯}{s}\in ]0,1]$ for Scenario III or $\overset{¯}{s}=1$ for Scenario II.

Based on the maximum $R^{2}$, the coefficients and parameters $\gamma_{0,opt}$, $\gamma_{1,opt}$, $\gamma_{2,opt}$, ${\overset{˜}{r}}_{opt}$, ${\overset{¯}{s}}_{opt}$ of the optimal model (Eq. (6)) were estimated. These values allowed us to define the relationship between pseudo prices $\overset{˜}{p}$ and actual prices *p* and consequently, ${\overset{˜}{r}}_{opt}$ and $r_{opt}$ (Eq. (7) and Eq. (8)):

(7) $$p_{it_{0}}=\gamma_{0,opt}+\gamma_{1,opt}log({\overset{˜}{p}}_{it_{0}})$$

(8) $$r_{opt}=\gamma_{1,opt}{\overset{˜}{r}}_{opt}$$

Finally, the accuracy of $r_{opt}$ and ${\overset{¯}{s}}_{opt}$ estimates was assessed by investigate the performance of the optimal model for values of *r* and $\overset{¯}{s}$ close to $r_{opt}$ and ${\overset{¯}{s}}_{opt}$.

## 3 Data

### 3.1 Market prices

Domestic price data were sourced from the Vulnerability Analysis and Mapping (VAM) Food and Commodity Prices Data Store of the UN World Food Program (Vulnerability Analysis and Mapping unit, 2014). In Senegal, VAM collects its data from the Commissariat pour la Sécurité Alimentaire. The data set consists of monthly retail prices (when available) from 60 markets distributed across the 14 regions of Senegal between 2012 and 2014. Of the four markets located in Dakar (Dakar, Tilène, Gueule Tapee and Castors), only the market with the least missing data (Tilène) was retained and used in the model.

**Figure 3:**
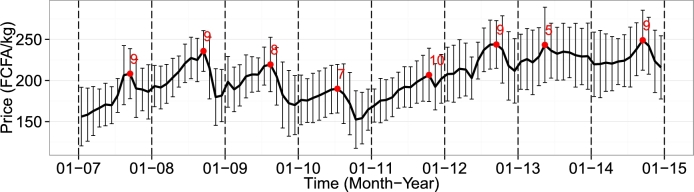
Average monthly millet prices (FCFA/kg) and standard deviation for each month from 2007 to 2014 in Senegal. Red annotations indicate the maximum value of each year and the corresponding month.

Figure 3 shows the temporal evolution of average millet prices in Senegal. A typical price decrease after harvest (around September) is clearly observable, followed by a gradual increase until the lean season. This trend is highly correlated with the supply trend. For instance, the impact of a poor and late harvest such as in 2011 (480 kt) was accompanied by a delay in peak price in October, compared with a relatively good year such as 2010 (810 kt) that is characterized by an early price decrease in July.

**Figure 4:**
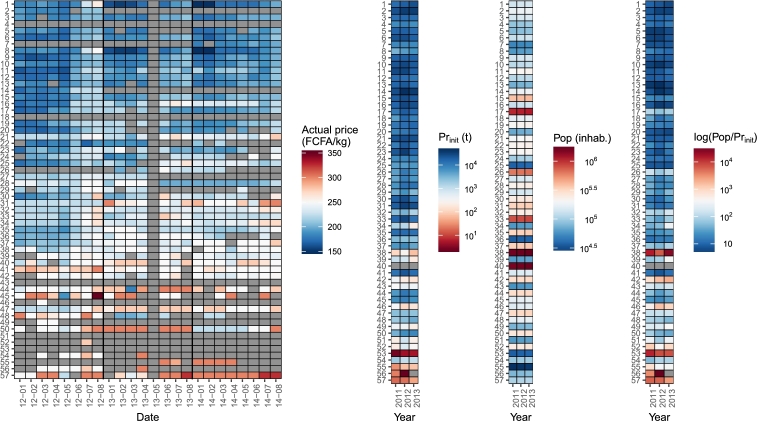
Millet prices, millet production, population and the ratio of population and production by market for 2012 to 2014 (January to August). Market numbers refer to the number-name matching in Figure 2.

A small price decrease at the beginning of some years (February 2008, February 2009, February 2010, March 2013) can be observed on Figure 3. It could be explained by rice substitution (harvested around October–December) resulting in a decrease of the demand for millet. Price data from May 2013 were discarded because they clearly exhibited errors in encoding or sampling (correlation with other months ranging from 0.05 to 0.35). Additionally, data for May 2012 were retained, even though the sudden drop was not unexplained. The spatial distribution of the price clearly shows a correlation with the production-population ratio (Figure 4).

Although not all markets had price data for the period of interest, we decided to keep them all to better simulate the spatial dynamics of market trade. Consequently, several price predictions were not validated due to missing data.

### 3.2 Catchment areas and transportation cost

Most of the food transportation (>95%) in Senegal relies on the road network (Bertholet et al., 2004). The distance by road was therefore used to approximate the transportation cost and define the catchment areas. A topological network was derived from the Global Insight data (manually edited by visual assessment in areas where important roads were missing) and minimum traveling times were computed using Dijkstra's algorithm (Dijkstra, 1959).

The transportation cost was defined as being directly proportional to the distance between markets (Eq. (2)). Transportation through the Gambia was assumed to be null, *i.e.*, the costs of crossing the border based on custom duties and other costs was assumed to be equivalent or more expensive than going around Gambia via the road. This assumption was consistent with the experience of local people.

The catchment areas for each market were estimated based on the area that minimizes the distance between each market (Figure 2). In the absence of secondary traveling directions, the underlying assumption was that traders travel to the nearest main road and then to the nearest market using the road network in order to sell their products. Each point in space was therefore assigned to a single market based on the shortest traveling distance by the nearest road.

### 3.3 Demand and population

Simplistically, the demand was estimated using population distribution maps acquired from the Worldpop project (Linard et al., 2012). A more realistic approach would have taken into account household income as well as taste and preferences but these data are generally unavailable at country level. Worldpop maps provide an estimate of the number of inhabitants in a given grid square (0.00083 decimal degrees, ∼100 m at the equator). The estimation was based on a random forest model trained on official 2009 population estimates at the commune level (method described in Stevens et al., 2015). The number of communes (113) was twice the number of catchment areas, ensuring accurate aggregation at the catchment level. The map for 2010 (not available for the years of interest) was used as a proxy of the spatial distribution of the population. The World Bank estimation of the total population for each year (2012–2014) was then used to adjust this distribution. The unequal birth rate throughout the country may affect the accuracy of this extrapolation.

### 3.4 Supply and production

In general, the production of cereal food crops is unable to meet the needs of the Senegalese population. Only in years with abundant rainfall does the country approach self-sufficiency in staple food crops in rural areas. Conversely, in times of poor harvest, millet is scarce due to the limited trade of this crop in the region. This deficiency is overcome by an increase of the rice imports, leading to a shift from millet to rice consumption in households that can afford it (Ndiaye, 2007). Most of the millet is produced in the regions of Kaolack, Kaffrine and Fatick in rotation with groundnuts, the main cash crop (Commissariat de la Sécurité Alimentaire, 2014). This rotation is crucial because groundnuts, being a legume crop, fix nitrogen in the soil.

Millet production estimates from the Direction de l'Analyse, de la Prévision et des Statistiques Agricoles (DAPSA) were selected as a proxy of the supply (Direction de l'Analyse, de la Prévision et des Stats Agricoles, 2013). These estimates are based on a two-stage stratified sample of around 6000 households and can be considered sufficiently accurate. However, because the granularity of these data is at the department level, 10-day temporal synthesis of 1-km SPOT-VEGETATION satellite images were used to convert them to the market catchment area level. The millet area was assumed to be uniformly distributed within the cultivated areas of a department, and the millet yield was spatially allocated to each 1-km pixel according to the distribution of Normalized Difference Vegetation Index (NDVI) data accumulated during the growing season. The NDVI, defined as the difference between near-infrared and red reflectances normalized by their sum, serves as a useful yield proxy when yield is mainly driven by plant vegetative growth, as occurs in regions where water or soil fertility are the main limiting factors, such as the Sahel (Rockström and De Rouw, 1997; Samaké et al., 2005).

In practical terms, cultivated areas were masked using the Land Cover Map produced by the Global Land Cover Network (2005; 1:100.000 scale; Leonardi, 2008) based on GlobCover 2005 map (Defourny et al., 2009), the most accurate map of the country (Waldner et al., 2015). Since we lacked reliable information on the spatial distribution of millet, we assumed that it was evenly grown across the cultivated area of a specific department. Then, for each pixel within cultivated areas, NDVI values above 0.2 during the millet growing season (from July to November) were integrated, which limited the contribution of bare soil to the signal. The actual millet production observed at the department level was then spatially allocated at the pixel level using the following equation (Eq. (9)):

(9) $$Prod_{k}=\frac{\sum_{t=jul}^{t=nov}NDVI_{k,t}}{{\sum^{n_{dpt}}}_{i=1}\sum_{t=jul}^{t=nov}NDVI_{i,t}}\times Prod_{dpt,k}$$

where $Prod_{k}$ is the estimated millet production for the pixel *k*, $NDVI_{k,t}$ is the NDVI above 0.2 for pixel *k* at time *t*, $Prod_{dpt,k}$ is the millet production from department *dpt* in which pixel *k* is located and $n_{dpt}$ is the number of pixels belonging to department *dpt*. Finally, using the market catchment area boundaries, pixel production values were aggregated to generate millet production by market.

### 3.5 Mobile phone calls, social capital and transaction costs

The last decade has seen a drastic increase of mobile phone users in Africa (Castells et al., 2009). In Senegal, the number of mobile cellular subscriptions was 25% in 2006 but reached 100% in 2014 (International Telecommunication Union, 2015). Several studies have demonstrated the impact of mobile phone access on price dispersion in food markets by, among others, reducing information costs (Aker, 2010; Jensen, 2007). Each time a call is made, a Call Data Record (CDR) is generated by the telecom companies for billing purposes. These metadata provide information on when, how and with whom one communicates. The communication itself is not recorded. After anonymization, some of these metadata were made available to the scientific community. As a result, the past few years have seen a rise in research projects such as the Data for Development challenge (www.d4d.orange.com) that was set up by Orange in 2013 (Ivory Coast) and 2015 (Senegal) to foster the use of CDRs for societal developments (de Montjoye et al., 2014). In particular, results have shown that the call intensity between people is a good indicator of social networks (Blondel et al., 2015; Candia et al., 2008; Eagle et al., 2008, Eagle et al., 2009). This property was used to approximate social capital assuming that business and social network were correlated.

The Call Data Records were provided by Sonatel Orange through the Data For Development (D4D) challenge framework. The original data set of phone calls between more than 9 million Orange customers in Senegal between January 1st, 2013 and December 31st, 2013 was processed to remove presumed machine-based calls or shared phones (see de Montjoye et al., 2014 for a description of the method).

From the mobile phone data, a contingency table with the yearly average sum of calls from, and to each market (from antenna within a buffer range of 10 km) was generated. This resulted in an origin-destination matrix containing the average number of calls between all market pairs. The transaction cost *s*, associated with social capital, was defined from this matrix following a two-step procedure.

First, the average number of calls made from and to each market pair was normalized by the calls made within the destination market, which was assumed to be proportional to the population living or working in the market area (Eq. (10)). This normalization was performed to ensure that small but close markets were considered well connected (Eq. (10)).

(10) $$Ncalls_{ij}=log\left(\frac{Calls_{ij}+Calls_{ji}}{Calls_{jj}}\right)\neq Ncalls_{ji}$$

where $Calls_{i,j}$ is the yearly average sum of calls from market *i* to market *j*. Ncalls provides an approximation of the strength of social relationships between traders of two markets.

Second, in order to translate Ncalls into *s*, we tested several limit values, $Ncalls_{lim}$, above which the markets were assumed to be perfectly connected (*s* = 1), and below which the markets were assumed to be perfectly isolated (*s* = 0), as follows (Eq. (11)):

(11) $$s_{ij}=\left\{ \begin{array}{c} 1\;\text{if }Ncalls_{ij}⩾Ncalls_{lim},\\ 0\;\text{ otherwise} \end{array}\right.$$

A value of $Ncalls_{lim}$ corresponds to a specific $\overset{¯}{s}$ value for all markets. Data were only available for 2013, we therefore assumed that the call pattern was similar for 2012 and 2014, since these are the two closest years.

## 4 Results & discussion

### 4.1 Scenario I (r=∞)

Panel I in Table 1 presents the results obtained by applying Eq. (4), *i.e.*, where all markets are independent. As expected, before transfers from surplus to deficit areas begin, the coefficient of determination between actual and estimated millet prices was low ($R^{2}=0.19\text{–}0.27$). This allowed us to reject the perfect market segregation scenario in Senegal. Adding the Month (*M*) variable improved the results for each year ($R^{2}=0.22\text{–}0.34$), particularly in 2012. This is explained by the higher temporal variation in prices for this year. The average price difference between January and August 2012 was 34.6 FCFA/kg, compared with 17.5 FCFA/kg in 2013 and 21.0 FCFA/kg in 2014. All coefficients were significant (p<0.01) and their expected sign was observed. Highly productive areas with low population tended to have lower prices than less productive areas with high population (positive coefficient) while the price tended to be higher in August than in January (positive coefficient). The coefficient of the Pop/Pr ratio was not significantly different from year to year.

**Table 1:** Multiple regression analysis results.

	Panel I – Eq. (4) for Scenario I with the month (w/ *M*) and without the month (w/o *M*) variable					
	2012 (n=354)		2013 (n=270)		2014 (n=299)	
	w/o *M*	w/ *M*	w/o *M*	w/ *M*	w/o *M*	w/ *M*
*α*_0_^a^	195.7 ± 3.0***	173.0 ± 4.0***	205.2 ± 3.4***	196.1 ± 4.7***	196.9 ± 3.1***	186.5 ± 4.4***
*α*_1_^a^ (*log*(*Pop*/*Pr*^*init*^))	6.3 ± 0.7***	6.2 ± 0.6***	6.4 ± 0.8***	6.3 ± 0.8***	7.0 ± 0.7***	7.0 ± 0.7***
*α*_2_^a^ (*M*)	–	5.0 ± 0.6***	–	2.1 ± 0.8**	–	2.4 ± 0.7**
*R*^2^	0.19	0.34	0.19	0.22	0.27	0.29


	Panel II – Eq. (6) for Scenario II and III					
	2012 (n=354)		2013 (n=270)		2014 (n=299)	
	Scenario II	Scenario III	Scenario II	Scenario III	Scenario II	Scenario III
*γ*_0,*opt*_^a^	77.9 ± 7.0***	−6.9 ± 10.0	−32.2 ± 14.0*	−71.9 ± 13.4***	72.1 ± 6.5**	63.6 ± 6.7***
*γ*_1,*opt*_^a^ (*log*(*Pop*/*Pr*^*eq*^))	36.5 ± 2.0***	62.1 ± 3.0***	84.4 ± 4.6***	97.0 ± 4.4***	43.2 ± 1.9***	46.0 ± 1.9***
*γ*_2,*opt*_^a^ (*M*)	4.8 ± 0.5***	4.7 ± 0.5***	2.0 ± 0.6***	2.3 ± 0.5***	2.8 ± 0.5***	2.8 ± 0.5***
*R*^2^	0.55	0.61	0.57	0.66	0.66	0.67
RMSE (FCFA/kg)	22.2	20.7	22.7	20.1	18.7	18.2


	Panel III – $r_{opt}$ and ${\overset{¯}{s}}_{opt}$ estimates					
	2012 (n=354)		2013 (n=270)		2014 (n=299)	
	Scenario II	Scenario III	Scenario II	Scenario III	Scenario II	Scenario III
*r*_*opt*_^b^ (FCFA/kg.100 km)	27.6 ± 4.1	23.2 ± 2.9	39.5 ± 3.3	31.8 ± 3.1	35.7 ± 3.8	34.6 ± 4.2
${\overset{¯}{s}}_{opt}$^b^	–	0.43 ± 0.09	–	0.43 ± 0.07	–	0.74 ± 0.08

a ± standard error.

b ± the error corresponding to the range of parameter values for which $R^{2}$ is not lower than 1% from the maximum $R^{2}$.

* p<0.1.

** p<0.01.

*** p<0.001.

### 4.2 Scenario II (0<r<∞ and $\overset{¯}{s}=1$)

**Figure 5:**
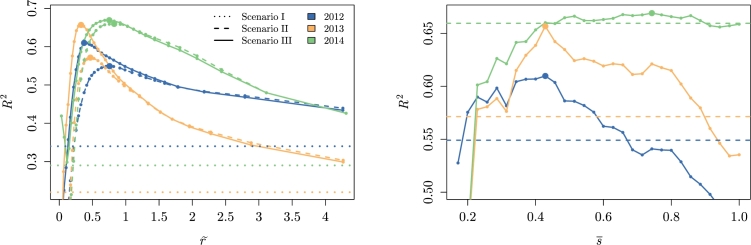
Coefficient of determination of the model prediction for several transportation pseudo cost values for Scenario I (*r* = ∞), Scenario II ($0<\overset{˜}{r}<\infty$ and $\overset{¯}{s}=1$) and Scenario III ($0<\overset{˜}{r}<\infty$ and $0<\overset{¯}{s}\leq 1$).

The situation with null transaction costs was studied thanks to the arbitrage condition defined in Eq. (2). The dashed lines on Figure 5 (left) show the $R^{2}$ of the model for several values of $\overset{˜}{r}\in \left[\text{min}\left(\frac{\Delta {\overset{˜}{p}}_{ij}^{init}}{d_{ij}}\right),\text{max}\left(\frac{\Delta {\overset{˜}{p}}_{ij}^{init}}{d_{ij}}\right)\right]$. Compared to the segregated markets situation (dotted lines on Figure 5, left), *r* alone was able to explain between 21 and 38% of the price variance between markets. The model converged to an optimal value, based on the maximum $R^{2}$, relatively similar from year to year (28–40 FCFA/kg.100 km, see panel III in Table 1).

These values of transportation costs define the trade flows based on differentials of $p_{t_{0}}$ which are not prices actually observed and should therefore not be directly interpreted. By comparison, transportation prices negotiated by smaller operators are ∼3–4.2 FCFA/kg.100 km (Hamilton, 2010). *r* takes into account more than just transportation costs, it also increases proportionally with distance. For instance, it is more risky to deal with remote traders, *e.g.*, due to lack of trust, or more expensive time investment. It is therefore very likely that social capital also explains part of the *r* value.

**Figure 6:**
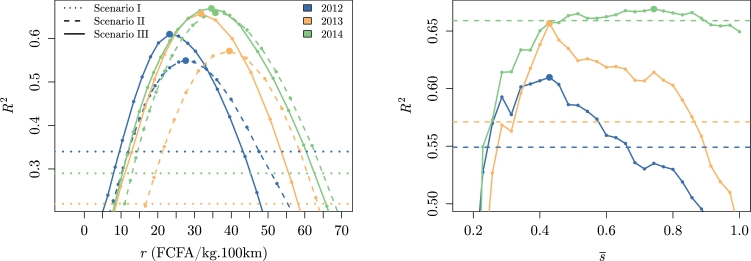
Coefficient of determination of the optimal model (using *γ*_0,*opt*_, *γ*_1,*opt*_ and *γ*_2,*opt*_) prediction for several transportation cost values for Scenario I (*r* = ∞), Scenario II ($0<\overset{˜}{r}<\infty$ and $\overset{¯}{s}=1$) and Scenario III ($0<\overset{˜}{r}<\infty$ and $0<\overset{¯}{s}\leq 1$).

The smoothness of the spatial equilibrium model convergence using $\gamma_{0,opt}$, $\gamma_{1,opt}$ and $\gamma_{2,opt}$ is shown on Figure 6 (left).

### 4.3 Scenario III (0<r<∞ and $0<\overset{¯}{s}\leq 1$)

When the transaction costs *s* were introduced, the performance of the model improved for each year ($R^{2}=0.61\text{–}0.67$, see the solid lines on Figure 5). The impact was higher for 2013, with 9% of the variance potentially explained by the transaction costs compared with 6% for 2012 and 1% for 2014 (see panel II in Table 1). It is also worth noting that, even though it was not its primary objective, the model was efficient to estimate prices, especially in light of the few parameters involved and the disparate nature of the data sources.

The performance of the model evolved as a function of $\overset{¯}{s}$ (Figure 5 and 6 on the right). The years 2012 and 2013 appeared to follow a similar trend (${\overset{¯}{s}}_{opt}∼0.4$), although with a different amplitude, whereas 2014 exhibited a better performance for lower transaction costs (${\overset{¯}{s}}_{opt}∼0.7$). Yet, the convergence appears noisy. This could be explained by the non-linearity of the spatial equilibrium model. At each iteration, 40 pairs of market were removed from trading (s=1 to s=0). It could therefore lead to abrupt changes within the optimization process. Furthermore, depending on the configuration of transaction costs, the production flow takes a preferential path that does not necessarily lead to linear change in $Pr^{eq}$ and therefore $R^{2}$. Having mentioned that, the trend remained clear but the accuracy of the optimum values must be interpreted with caution.

From these results, the impact of social capital on millet prices (regardless the distances between markets), approximated by the business network between agents of different markets, was clearly demonstrated. Transaction costs, as defined in this study, reflect the business network of people working in a given market. Trading is easier between trustworthy people belonging to the same social network. In the model, a high *s* prevents a trader from directly taking advantage of a profitable arbitrage opportunity. It forces the trade flow to follow a less risky path. In other words, *s* determines the preferential flow path of trade. Transaction costs might also reflect heterogeneous *r* that could be related to a lack of local competition, or differences in road quality. However, the fact that *s* is asymmetric mitigates this hypothesis.

The specificity of the impact of social capital, in particular the difference between the years, is challenging to assess because $Pr^{eq}$ –the only variable that changes in the model– depends on the interaction between $Pr^{init}$, *r*, *s* and *d*. The combination of these four parameters determines the production flow and ultimately, $Pr^{eq}$. Furthermore, the validation data set was incomplete (Figure 4) which could result in local over-fitting and flaws in the interpretation of the results.

In light of theses observations, one should not jump to any conclusion on the year-specific impact of the transaction costs. The agricultural production in the 2011/2012 harvest was lower than usual (∼480 kt), due to late onset of the rainy season, dry spells, early cessation of rains, and late provision of inputs (World Food Programme, 2012). In comparison 2012/2013 (∼660 kt) and 2013/2014 (∼515 kt) were average years. The higher effect of social capital on 2012 and 2013 millet prices suggests that risk aversion would be strengthened following a poor harvest with an effect that stands for two years. It could also be that 2014 is a specific year during which social capital has few impact on millet prices. Additional data from other years would be needed to draw any conclusion on this point. It should also be mentioned that mobile phone data used to approximate social capital only covers 2013, the year with the highest effect of social capital on prices. While it is unlikely that social network radically changes from year to year, small changes can have an great impact if there are focused on important market pairs.

### 4.4 Residuals

Based on the specific values of the residuals, May 2012 was systematically overestimated (+9.8 FCFA/kg in average) due to unexpectedly low actual price values (Figure 4). Discarding this month yielded significant improvement in the model fit ($R^{2}=0.64$, n=308) for the same $r_{opt}$ and ${\overset{¯}{s}}_{opt}$ values.

The largest residuals (absolute value > 0.2) generally corresponded to unexpected values in the price database, such as the price in Thiodaye for May and August 2012, or in Bignona for February 2012 (Figure 4). These outliers could be explained by either sampling or encoding errors, or market failures. In the latter case, the difference between the actual and predicted price could then be used as an indicator of market failures associated with unexpected events such as religious feasts, storage effects, food aid or adequate substitution.

Some of the markets associated with the outliers were also poorly estimated, suggesting that their under or overestimation could be explained by systematic inaccuracies for these markets. This could be due to inaccuracies in the delineation of the catchment area, resulting in poor estimation of local production and population.

### 4.5 Trade flows

**Figure 7:**
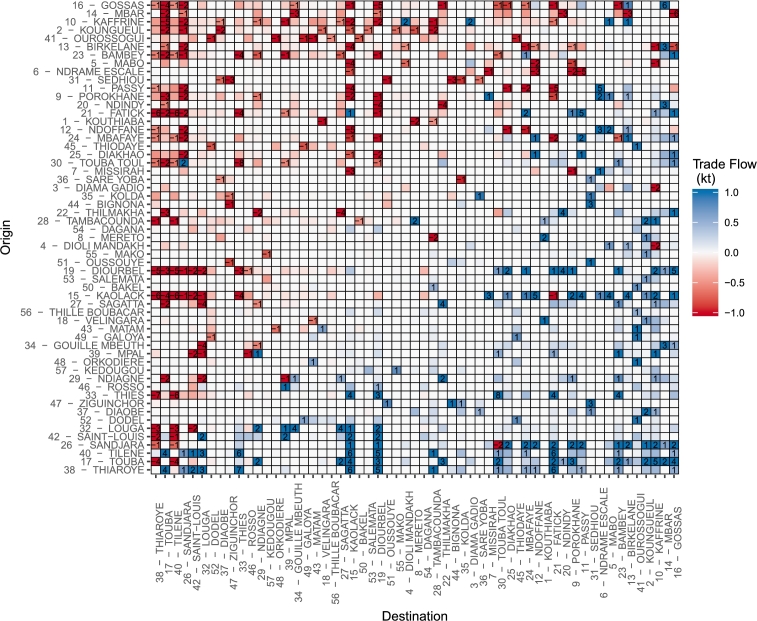
Average net trade flows (computed in kt of millet production), for all years, between all market pairs. Numbers on trade flows maps indicate flows with higher intensity than 1 kt.

The average trade flows can be used to classify the markets into sinks and sources of production (Figure 7). Gossas (16; the numbers between brackets refer to market number-name matching from Figure 2), Mbar (14) and Kaffrine (10) were the main sources of millet production, and Tilène (40), Thiaroye (38) and Touba (17) were the main sinks. Unsurprisingly, these corresponded to the main production and populated areas, respectively. Most interesting were intermediary situations involving assembly markets such as Kaolack (15) or Diourbel (19) that had similar inflows and outflows. These markets are believed to be critical for the functioning of national trade. Interestingly, a great number of market pairs did not exchange any goods, but no market was completely isolated, *i.e.*, with null flows. Trade flow was not necessarily direct between origin and destination markets, since production could transit through intermediary markets. Therefore, markets with null flow might temporarily host some production that was subsequently transported to another market until gradually reaching its final destination. Such intermediary transits were not described by the model. Caution should therefore be taken when interpreting flow values, since they may not correspond to actual estimation of production flows which are challenging to validate, but rather to an indicator of trade intensity between two markets.

**Figure 8:**
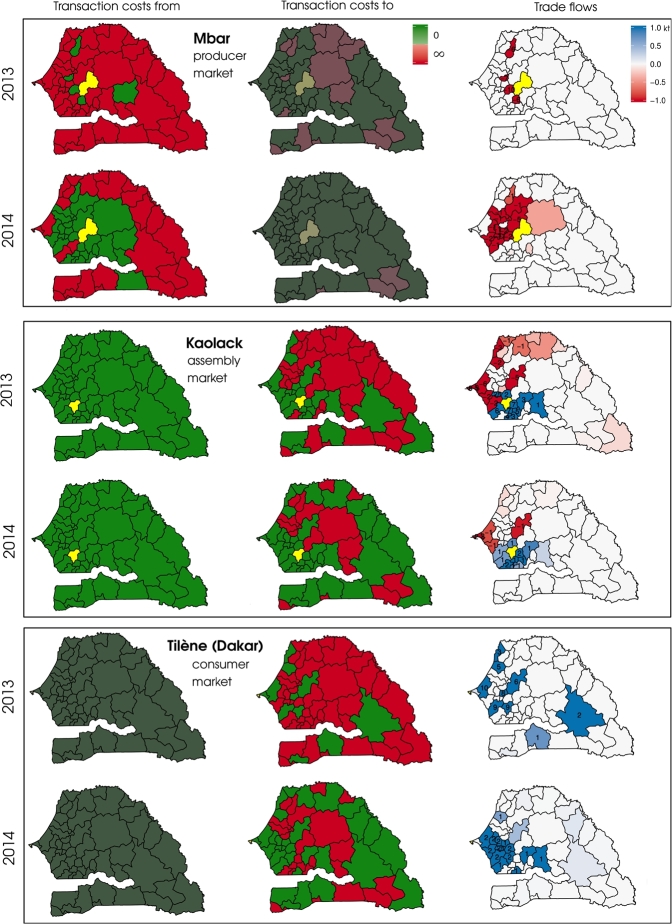
Trade flows of, and transaction costs, *s*, from and to the markets (in yellow) of Mbar (14, Producer), Kaolack (15, Assembly) and Tilène (40, Consumer) in 2013 ($\overset{¯}{s}=0.43$) and 2014 ($\overset{¯}{s}=0.74$). Numbers on trade flows maps indicate flows with higher intensity than 1 kt. Transaction costs maps in dark color for Mbar and Tilène should not be interpreted as the trade flow uses only one direction for these markets.

As an illustration, Figure 8 shows the impact of the transaction costs on the trade flows for three main markets in 2013 and 2014. Flows in 2012 were very similar to 2013 but lower in intensity.

According to our model, Mbar (14) is a major producer market and one of the main sink of production. Therefore, the integration *from* Mbar *to* the other markets drove the trade flows. In 2014, the trade possibilities were numerous and around half of them led to transfers of millet. The other half were markets which were either located in distant regions or associated with a low population. In 2013, trade opportunities were very limited and, as a result, the intensity of flows was higher and more concentrated. Interestingly, the market of Ndindy (20) and Birkelane (13) received 7 kt and 6 kt in 2013 from Mbar, but nothing in 2014. This clearly illustrates the non-linearity of the trade flows. A high quantity of trade does not necessarily imply that the receiving markets were the final destinations. On the contrary, in 2013, it is likely that a large part of the production was re-transferred to other markets, eventually reaching the same markets than 2014 but using indirect routes. However, taking longer routes leads to market inefficiency in production distribution and therefore, price transmission.

Kaolack (15) is one of the most important assembly markets of the country. It collects the millet production from the cropping areas and distributes it to consumer markets. This role was accurately described by our model: millet was transferred from several rural areas to Kaolack and consumer markets received production from Kaolack. This role seems to be exacerbated when the transaction costs are high such as in 2013 because this market remained well connected to the rest of the country even in case of low $\overset{¯}{s}$. It even shipped production to remote markets such as Kedougou (57).

Finally, Tilène (40), the market in Dakar, draws in production from the interior of the country. This market is a good example of the impact of *s* in the model. In 2013, net flows were distributed to almost all markets trading with Dakar. Since these markets also received production from producer markets, it does not entail that the millet they sent to Dakar was *produced* in their own catchments areas. Rather, production was successively transported from one market to the other before reaching these areas. Unsurprisingly, Kaolack (15), Diourbel (19), Thiès (33) and Touba (17), the four major assembly markets, were among these markets. In 2014, net flows appeared to come from closer markets, reflecting the smaller trade constraints of that year.

### 4.6 Limitations

Lacking data on self-consumption, we made the hypothesis that all the millet production was available for trade or that the individual self-consumption was similar everywhere. Millet was also assumed to be planted evenly within the cropland, which is inaccurate. However, the aggregation of production at catchment area level is likely to mitigate the effects of this simplification. The demand function would have been estimated more realistically if it accounted for household income as well as taste and preferences but these data are generally unavailable at country level. Furthermore, substitution effects with other crops such as rice were not considered.

The roads data were checked and edited manually so that all major access routes were taken into account. The speed of these roads (due to legal limitations or quality) was unknown and therefore neglected. This could lead to an underestimation of the catchment areas of urban markets (or the opposite for rural markets) by underrating their attractiveness since they are often equipped with better roads compared to rural markets. Besides, historical and cultural preferences were not taken into account in the definition of catchment areas. Additionally, transport by rail or boat was not included since neither are used extensively for millet transportation (Bertholet et al., 2004). Finally, inter-border trade was not considered but it occurs only marginally due to the constraints of international trade for small producers and the weak import demand for millet (Ndiaye and Niang, 2010).

When using CDR data to analyze the whole population, several bias might arise and limit the generalization of the analysis. First, we assumed that every agent (at least every traders) owns a mobile phone. In 2013, there were 93 mobile phone subscriptions per 100 inhabitants which implies that most of the Senegalese population owns cell phones (ITU World Telecommunication, 2016). However, by using only CDR data, some demographic sub-groups, in particular the poorest, are still left out the analysis. This means that the social network of poorer traders could have been underrated. Second, Sonatel's market share reached nearly 62% of the cell phone market in 2013 which entails that a selective bias may arise from the different demographic groups targeted by each operator (Autorite de Régulation des Telecommunications et des Postes, 2013).

We selected phone calls as the proxy of the business network between two market areas. This choice relies on the hypothesis that the social network and business network are similar as business calls are indistinguishable from the others. In doing so, some business ties could have been overestimated when the social network is stronger than the business network between two market areas, and the other way around. For the sake of simplicity, the transaction costs associated with social capital were defined as a dummy variable, *i.e.*, null or infinite depending on a threshold on the number of calls. However, a large range of situations exists between these two extreme cases and could have been considered by rewriting Eq. (3) as Eq. (12).

(12) $$p_{i}-p_{j}\leq d_{ij}r+f(s_{ij})$$

with f(.) some increasing and convex cost function.

### 4.7 Policy implications

Several policies could be put in place to cut down transportation costs, the main source of inefficiency in millet markets. As pointed out previously by Teravaninthorn and Raballand (2009), it is not so much that transportations costs in Africa are higher than those in other developing regions such as China, but transportation prices are much higher. Administrative barriers are at least as important as poor roads in hindering the market functioning, particularly in western and central Africa. Removing restrictions on the entry of new companies into the market should stimulate competition and reduce the high profits of local trucking companies. Rwanda is a well-known example of an African country that deregulated its transport sector and saw a dramatic drop in transport prices almost overnight (Teravaninthorn and Raballand, 2009). The trucking industry in Senegal is dominated by a large number of very small operators who own and operate an obsolete trucking fleet (Hamilton, 2010). Improving infrastructure and the trucking industry are therefore expected to have a major impact on market functioning and prices.

Understanding the role that social capital plays in market exchanges is essential for policy design. Finding approaches to facilitate search and fostering trust will likely improve trade exchange. The only gateway for policymaker is to work on social structures via formal institutions (*e.g.*, legal institution, public market information system) or interpersonal relationships (*e.g.*, fostering traders' associations or the learning of different languages). Functioning institutions and strong governance make transactions impersonal leading to economic efficiency (Rashid et al., 2010; Durlauf and Fafchamps, 2005). Law and court should therefore be strengthened especially in poor countries where many transactions are small and buyers and sellers are too poor for court action to yield reparation (Bigsten et al., 2000; Fafchamps and Minten, 2002). Whether or not social capital simplifies market trade is an indicator of the efficiency and reach of formal institutions.

## 5 Conclusions

In this study, we demonstrated the effect of social capital on millet prices in Senegal. Social capital was approximated by the business network of economic agents using a unique data set on mobile phone communications between 9 million people. Our approach was a spatial equilibrium model that accounts for both transportation (*r*) and transaction costs (*s*) and successfully estimated millet prices in 57 markets in Senegal for three contrasted years.

The transportation costs were modeled proportionally to the distance and accounted on average for the majority of price differentials in the country (∼20–40%). Clearly, this is the main source of inefficiency in millet markets in Senegal. Several components of freight cost are probably included in this value such as maintenance, opportunities, etc. Other transaction costs proportional to the intermarket distance could also be involved but they remain challenging to isolate. In particular, the impact of social capital on market functioning could already be accounted for in this value due to, for instance, mistrust in remote traders.

The transaction costs, modeled as null or infinite, accounted for between 1 and 9% of the price variance, demonstrating the effect of social capital on millet prices. Interpreting *s* and its specific impact is not straightforward and remains challenging to validate. The impact of *s* is marked for two years following a poor harvest, *e.g.*, in 2012 and 2013. In this situation, the assumption is that events result from traders managing their risk by focusing their commercial transactions on well-known and therefore less risky markets, *i.e.*, the aversion to risk is higher following a poor production. However, additional data from other years are still required before reaching firm conclusions on this point.

This work opens new avenues for (i) research on social capital and market integration, (ii) better integration of the two first pillars of food security, *i.e.*, availability of and access to food, and (iii) more comprehensive implementation of early warning systems for food security in the region. Further insights can be expected from expanding the model to other countries in the Sahel as well as exploiting multiple years of mobile phone data.

## Declarations

### Author contribution statement

Damien Jacques: Conceived and designed the experiments; Performed the experiments; Analyzed and interpreted the data; Contributed reagents, materials, analysis tools or data; Wrote the paper.

Eduardo Marinho: Conceived and designed the experiments; Analyzed and interpreted the data.

Francois Waldner, Raphael d'Andrimont, Julien Radoux: Contributed reagents, materials, analysis tools or data; Wrote the paper.

Frederic Gaspart, Pierre Defourny: Contributed reagents, materials, analysis tools or data.

### Funding statement

This work was partly supported by the Belgian National Fund for Scientific Research through a FRIA grant (5211815F), and the Bill & Melinda Gates Foundation (OPP1114791).

### Competing interest statement

The authors declare no conflict of interest.

### Additional information

No additional information is available for this paper.
